# Beyond facilities, policy implications of the nursing home for senior citizens act

**DOI:** 10.1017/S1478951526101680

**Published:** 2026-01-29

**Authors:** Richard Ian Mark T Necosia, Joanne Vivien B. Necosia, Joy Molina Mirasol, Don Eliseo III Lucero-Prisno

**Affiliations:** 1 College of Public Administration and Governance, Bukidnon State University, Bukidnon, Philippines; 2 College of Arts and Sciences, Bukidnon State University, Bukidnon, Philippines; 3 Department of Global Health and Development, London School of Hygiene & Tropical Medicine Faculty of Public Health and Policy, London, UK

Dear editor,

Layne et al. (2026) present timely evidence on the feasibility and acceptability of early palliative care for persons living with dementia and their caregivers through the SUPPORT-DTM program. Their findings are relevant not only to clinical practice but also to policy debates in countries that are expanding institutional care for older persons.

In the Philippines, the proposed Nursing Home for Senior Citizens Act seeks to establish at least one nursing home in every city or province to provide safe and affordable care for older adults. The bill responds to demographic aging, family strain, and limited geriatric services. Its provisions emphasize facilities, custodial care, medical consultation, counseling, and social activities. These are essential components. Yet the legislation risks framing elder care primarily as an infrastructure concern unless psychosocial and palliative dimensions are explicitly strengthened.

Dementia care illustrates this policy gap. As shown in the SUPPORT-DTM study, emotional distress, caregiver burden, and planning challenges emerge early in the disease trajectory, long before institutional admission becomes necessary (Layne et al. 2026). Nursing homes that prioritize accommodation and basic medical monitoring may intervene too late, after opportunities for anticipatory guidance, dignity preservation, and shared decision making have diminished. Early palliative care addresses these needs upstream, where policy impact is greatest.

This issue must also be viewed within the broader Philippine health system context. Health services have historically received limited prioritization in national budgeting, with preventive, mental health, and community-based care often underfunded. Establishing nursing homes without sustained investment in health and palliative services risks creating facilities that are physically present but clinically and psychosocially under resourced. For conditions such as dementia, this gap may translate into custodial care that manages decline rather than supports meaning, comfort, and family well-being.

The SUPPORT-DTM model offers concrete lessons for policymakers. Its nurse led and structured approach aligns with workforce realities in resource constrained settings. The modest changes observed in some outcomes should not be interpreted as limited value. In progressive illness, maintaining stability in stress, quality of life, and caregiver burden constitutes a meaningful outcome, particularly when compared with crisis driven care and late-stage institutionalization (Layne et al. 2026).

Senate Bill No. 20 provides an opportunity to move beyond facility-based solutions. Nursing home legislation should explicitly require early palliative and psychosocial care as core services, supported by adequate health financing and clear implementation standards. Without this policy shift, nursing homes risk becoming custodial spaces rather than settings of dignity, compassion, and shared responsibility for older persons and their families.

## References

[ref1] Layne D, Kelechi T, Milano N, et al. (2026) A program of SUPPORT-DTM: feasibility and acceptability of an early palliative care intervention for those living with dementia and caregivers. *Palliative & Supportive Care* 24(e20), 1–9. doi:10.1017/S1478951525101429 PMC12926733 41498161

